# miR-15b-5p induces endoplasmic reticulum stress and apoptosis in human hepatocellular carcinoma, both *in vitro* and *in vivo*, by suppressing Rab1A

**DOI:** 10.18632/oncotarget.3970

**Published:** 2015-05-18

**Authors:** Yang Yang, Ni Hou, Xiaofei Wang, Lumin Wang, Su'e Chang, Kang He, Zhenghao Zhao, Xiaoge Zhao, Tusheng Song, Chen Huang

**Affiliations:** ^1^ Department of Genetics and Molecular Biology, Xi'an Jiaotong University College of Medicine, Xi'an, Shaanxi, China; ^2^ Key Laboratory of Environmentally and Genetically Associated Diseases at Xi'an Jiaotong University, Ministry of Education, Xi'an, Shaanxi, China; ^3^ Cardiovascular Research Center, Xi'an Jiaotong University College of Medicine, Xi'an, Shaanxi, China

**Keywords:** miR-15b-5p, hepatocellular carcinoma, Rab1A, apoptosis, ERS

## Abstract

In human hepatocellular carcinoma (HCC), aberrant expression of miRNAs correlates with tumor cell proliferation, apoptosis, invasion, and migration by targeting downstream proteins. miR-15b levels are reported increased in HCC tissues; however, they negatively correlate to HCC recurrence. Our aim was to understand the reason for this phenomenon. We used the reverse transcription-polymerase chain reaction (RT-PCR) to measure miR-15b-5p expression in both HCC tissues and HCC cell lines. Our results were consistent with the report. Using bioinformatics and luciferase reporter assays, we identified Rab1A as a novel and direct target of miR-15b-5p. Inhibiting the function of Rab1A with shRab1A also inhibited the growth of HCC cells and induced endoplasmic reticulum stress (ERS) and apoptosis. Similarly, suppressing Rab1A by overexpression of miR-15b-5p also inhibited cell growth and induced ERS and apoptosis. Moreover, re-expression of Rab1A rescued the miR-15b-5p -induced ERS, apoptosis, and growth inhibition in HCC cells. *In vivo* assays were further performed to testify them. Taken together, our data suggest that miR-15b-5p induces ERS, apoptosis, and growth inhibition by targeting and suppressing Rab1A, acting as a tumor suppressor gene in HCC. This finding suggests a novel relation among Rabs, miRNAs, and apoptosis.

## INTRODUCTION

Human hepatocellular carcinoma (HCC) is one of the most common cancers worldwide. HCC ranks as the third leading cause of cancer-related deaths globally, with a poor prognosis of limited survival [1]. Currently, treatment for HCC includes surgery, systemic or local chemotherapy, radiation therapy, multimodality therapy, or a combination of therapies [2]. Unfortunately, approximately 80% to 90% of patients with HCC are not eligible for surgical intervention because of the difficulty of effectively diagnosing HCC at early stages [3, 4]. Thus, new treatment approaches are needed.

MicroRNAs (miRNA) are an abundant class of noncoding RNAs, ranging from 19 to 24 nucleotides. They are evolutionarily conserved and function as regulators of gene expression by targeting mRNAs for cleavage or translational repression [5]. Bioinformatics analysis suggests that approximately one-third of the human protein coding genes, particularly classical oncogenes or tumor suppressor genes, are regulated by miRNAs. [6, 7]. miRNA dysfunction is associated with a variety of human diseases, including cancer, suggesting that miRNAs play pivotal roles in cancer [8]. In HCC, aberrant expression of miRNAs, including miR-195, miR-214, and miR-302b suppresses tumor cell proliferation, apoptosis, and invasion by targeting downstream proteins [9–11]. miR-15b, a member of the miR-16 family(miR15a/b, miR-16, miR-195, miR-424 and miR-497), which targets genes important for the G1 - S transition, is reported increased in HCC [12]. Nevertheless, miR-15b negatively correlates with HCC recurrence despite the ability to inhibit HCC cell proliferation. The mechanism behind this discrepancy remains unclear.

Rab proteins are a large family of small GTPases that function directly in intracellular vesicular trafficking. There are at least 60 members of the Rab family in the human genome [13]. Rab proteins are localized on specific intracellular compartments where they participate in distinct steps in membrane trafficking pathways. [14]. They work as molecular switches; with their structures cycling from the inactive GDP-bound states to the active GTP-bound states [15, 16]. Rab proteins regulate membrane transport and impact cell signaling pathways by binding to effecter proteins. As a member of the Rab family, Rab1A acts as a Golgi-resident Rab to control vesicle trafficking from the endoplasmic reticulum (ER) to the Golgi apparatus [17, 18]. Inhibition of Rab1A function results in the accumulation of protein within ER, which induces an ER stress (ERS) signaling pathway referred to as the unfolded protein response (UPR). UPR coordinates protein synthesis and degradation, chaperone expression, cell cycle progression, and apoptosis. [19]. If UPR is not effective in reducing ERS, cell apoptosis is induced.

In this study, we found that miR-15b-5p was frequently increased in HCC tissues and cells, which is consistent with the previous findings [20]. Earlier studies demonstrated the negative correlation of miR-15b with HCC recurrence [21] which indicates that miR-15b-5p may act as a tumor suppressor in cancer progression even though it is increased in HCC. However, the underlying mechanism still remains unclear. Experiments performed *in vitro* demonstrated that the overexpression of miR-15b-5p significantly inhibited proliferation and induced apoptosis of HCC cells. Bioinformatics analysis and dual-luciferase reporter assays indicated that Rab1A is a novel target of miR-15b-5p. Inhibition of Rab1A by shRab1A induced ERS and apoptosis. Likewise, we found that, in addition to inducing ERS and apoptosis, suppressing Rab1A by over-expressing miR-15b-5p or shRab1A suppresses the growth of SMMC-7721 cells and Hep3B cells, both *in vivo* and *in vitro*. Taken together, we suggest that miR-15b-5p induces ERS and apoptotic death in HCC cells by targeting and suppressing Rab1A.

## RESULTS

### miR-15b-5p is significantly increased in HCC tissue samples and HCC cell lines

**Figure 1:**
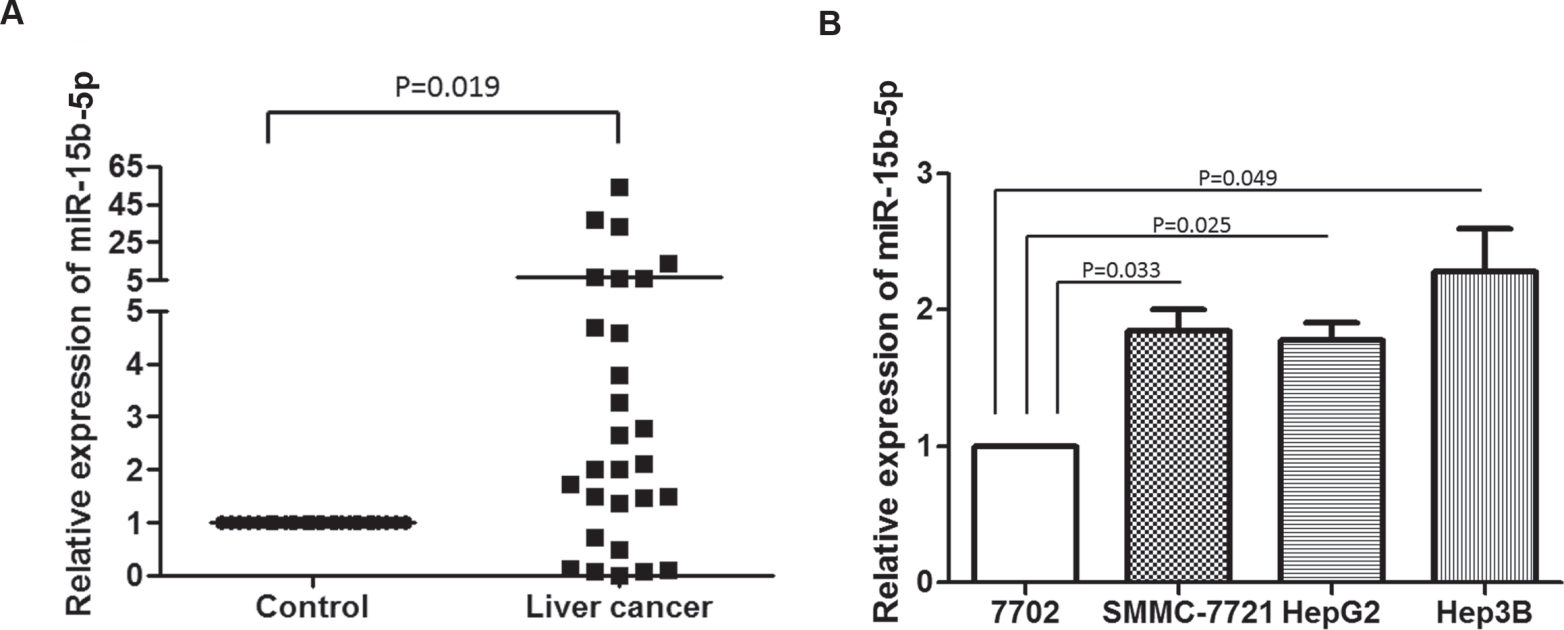
miR-15b-5p was increased in HCC tissues and HCC cell lines **A.** miR-15b-5p expression levels were detected in 28 paired HCC tissues and their normal control tissues using qRT-PCR. **B.** Relative levels of miR-15b-5p expression in the HCC cell lines. The expression of miR-15b-5p was normalized to U6. Data represented the mean ± SEM.

To analyze the expression of miR-15b-5p in liver cancer, qRT-PCR was used to measure the expression of miR-15b-5p in 28 paired HCC and matched normal tissues. The expression of miR-15b-5p was high in most HCC tissues (Figure 1A). We also examined the expression of mature miR-15b-5p in three HCC cell lines and in a normal liver cell line, HL-7702. The expression of miR-15b-5p in the three HCC cell lines was significantly higher than in the HL-7702 cells (Figure 1B). These results indicated that miR-15b-5p was significantly increased in HCC tumor tissues and HCC cell lines.

### miR-15b-5p directly targets and suppresses Rab1A

**Figure 2:**
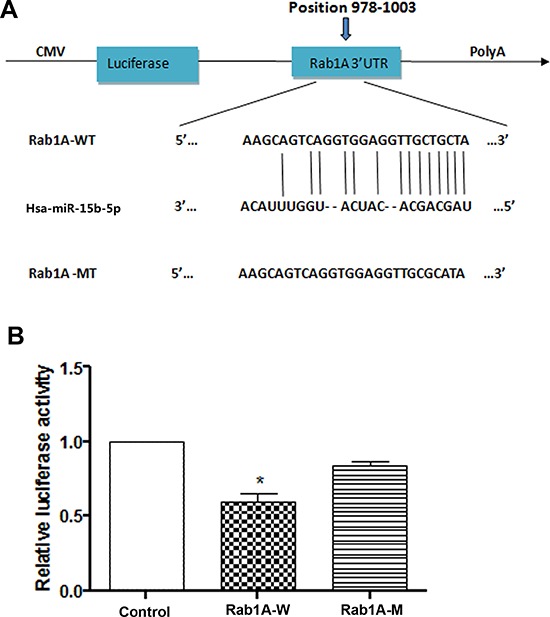
miR-15b-5p directly targeted Rab1A **A.** Scheme of the potential binding sites of miR-15b-5p in the Rab1A 3′ UTR. **B.** Luciferase assay in HEK293 cells. Pre-miR-15b was co-transfected with pGLO-Rab1A –wt 3′ UTR or pGLO-Rab1A –mut 3′ UTR or control. Luciferase activity were measured 24 h after transfection, a significant decrease in pGLO-Rab1A group was displayed. Data represented the mean ± SEM (*p < 0.05; **p < 0.01).

Three different programs were used to identify the molecular targets of miR-15b-5p. Because miRNAs can post-transcriptionally inhibit target mRNAs by binding to the 3′-UTR, Rab1A was identified as a potential target of miR-15b-5p based on sequences in the 3′-UTR that were complementary to the seed sequence of miR-15b-5p. Moreover, we found that the seed sequences were evolutionarily conserved (Figure 2A). To experimentally determine if miR-15b-5p directly targets Rab1A, dual luciferase reporter assays were performed. HEK293 cells were co-transfected with miR-15b-5p and either Rab1A-wild type (wt) 3′-UTR or Rab1A-mutant (mut) 3′-UTR vector. Cells were transfected with pmirGLO vector as the control. The cotransfection of miR-15b-5p with Rab1A-wt 3′-UTR, but not with Rab1A-mut 3′-UTR, resulted in significant downregulation of luciferase activity than that in the transfection with control vector (*P* < 0.05) (Figure 2B). To measure protein expression, western blot analysis was performed using lysates from SMMC-7721 and Hep3B cell. Western blot analysis indicated that miR-15b-5p decreased Rab1A expression at the protein level in both SMMC-7721 and Hep3B cells and that inhibition of miR-15b-5p significantly increased the protein levels of Rab1A (Figure 4C). In addition, as shown in Supplementary Figure S1, we found there was a inverse correlation between miR-15b-5p and Rab1A mRNA expression levels. These results suggest that miR-15b-5p directly targets and suppresses Rab1A.

### miR-15b-5p inhibits the growth of HCC cells by targeting and suppressing Rab1A

**Figure 3:**
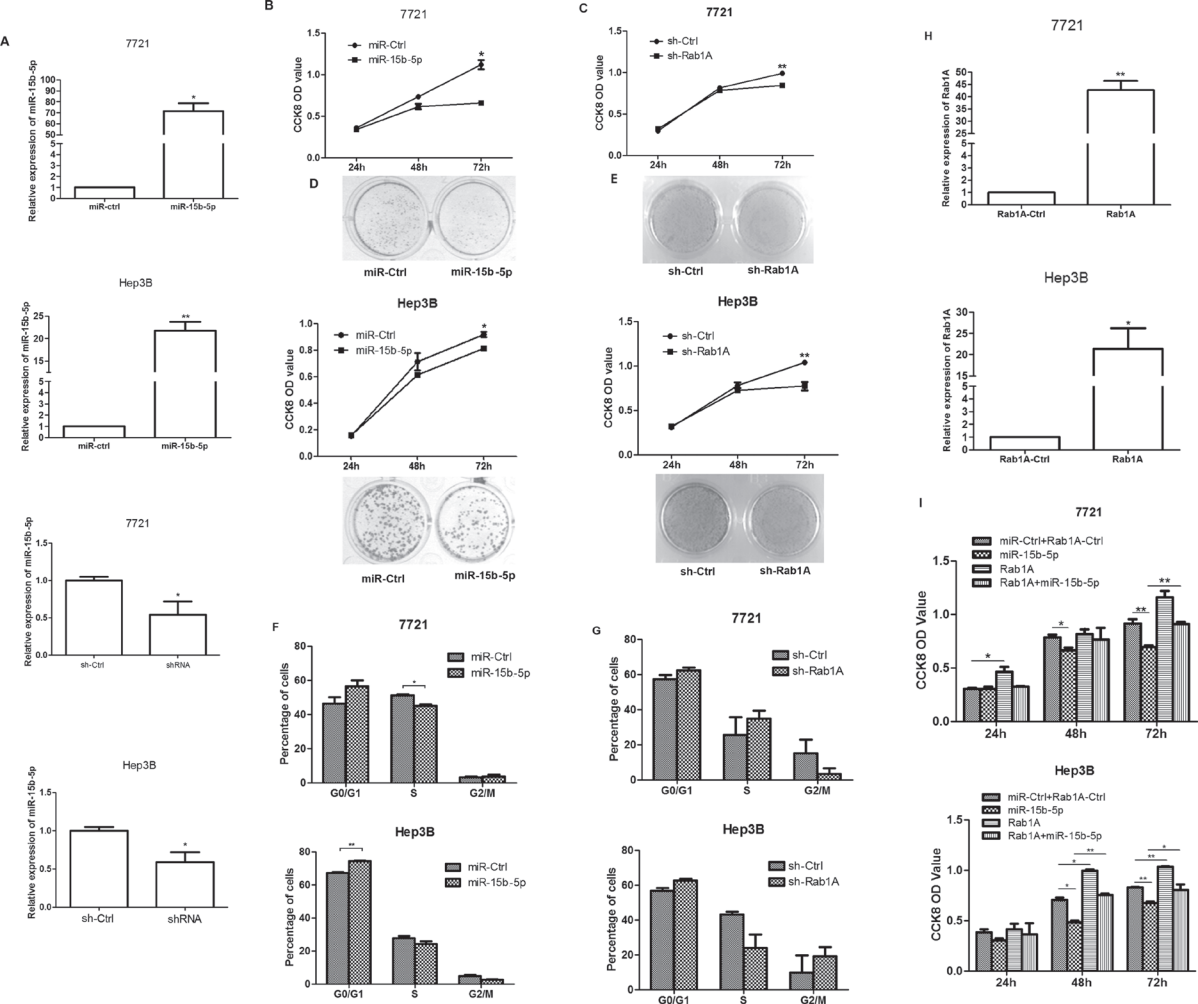
miR-15b-5p suppressed HCC cell growth by targeting and suppressing Rab1A **A.** The expression of miR-15b-5p was significantly increased after transfected with miR-15b-5p in SMMC-7721 and Hep3B cells. sh-Rab1A could reduced the expression of Rab1A significant in both SMMC-7721 and Hep3B cells. **B.** The effects of miR-15b-5p on SMMC-7721and Hep3B cell proliferation were determined by CCK-8 assay after transfection for 24, 48, and 72 hours with miR-15b-5p or control vector, respectively. **C.** SMMC-7721 and Hep3B cells proliferation determined by CCK-8 assay at 24, 48 and 72 h after transfection with shRab1A or control vector. **D-E.** colony formation assay was also performed to detect the effect after transfected with miR-15b-5p or shRab1A with their control, respectively. **F-G.** The representative histograms for cell-cycle distribution transfected of miR-15b-5p or shRab1A for 48 h in both SMMC-7721 and Hep3B cells. **H-I.** Overexpression of Rab1A reverses the anti-proliferative roles of miR-15b-5p. Data were presented as mean ± SEM (*P < 0.05; **P < 0.01.).

The expression of miR-15b-5p was significantly increased after transfected with miR-15b-5p in SMMC-7721 and Hep3B cells (Figure 3A). In an attempt to explore the effects of miR-15b-5p on the growth of HCC cells by targeting and suppressing Rab1A, we purchased shRab1A and its control from GeneChem, (Shanghai, China), which induced about 50% decrease of Rab1A expression at the mRNA levels in SMMC-7721 cells and Hep3B cells (Figure 3A). Cells were transfected with miR-15b-5p or sh-Rab1A. The CCK8 assay indicated that cell growth was inhibited in SMMC-7721 and Hep3B cells transfected with miR-15b-5p or sh-Rab1A (Figure 3B-3C). The CCK8 assay indicated that cell growth was inhibited in SMMC-7721 and Hep3B cells transfected with miR-15b-5p or sh-Rab1A. To further examine the inhibitory role of miR-15b-5p, colony formation assay was performed. Notably, fewer and smaller colonies were observed in cells transfected with miR-15b-5p or shRab1A than those transfected with control (Figure 3D-3E). A cell cycle assay was performed to detect the percentage of cells in the G1, S, and G2 phases of the cell; however, no change was detected (Figure 3F-3G). To further demonstrate the connection between miR-15b-5p and Rab1A, SMMC-7721 or Hep3B cells were further transfected with the Rab1A expression vector or the control vector after transfection of miR-ctrl or miR-15b-5p. We purchased Rab1A and its control from GeneChem, (Shanghai, China), which significantly increased Rab1A expression at the mRNA levels in SMMC-7721 cells and Hep3B cells (Figure 3H). The re-expression of Rab1A reversed the growth inhibition that was induced by miR-15b-5p (Figure 3I). These data suggest that miR-15b-5p suppresses the growth of HCC cells by targeting and suppressing Rab1A.

### miR-15b-5p induces HCC cells apoptosis by targeting and suppressing Rab1A

**Figure 4:**
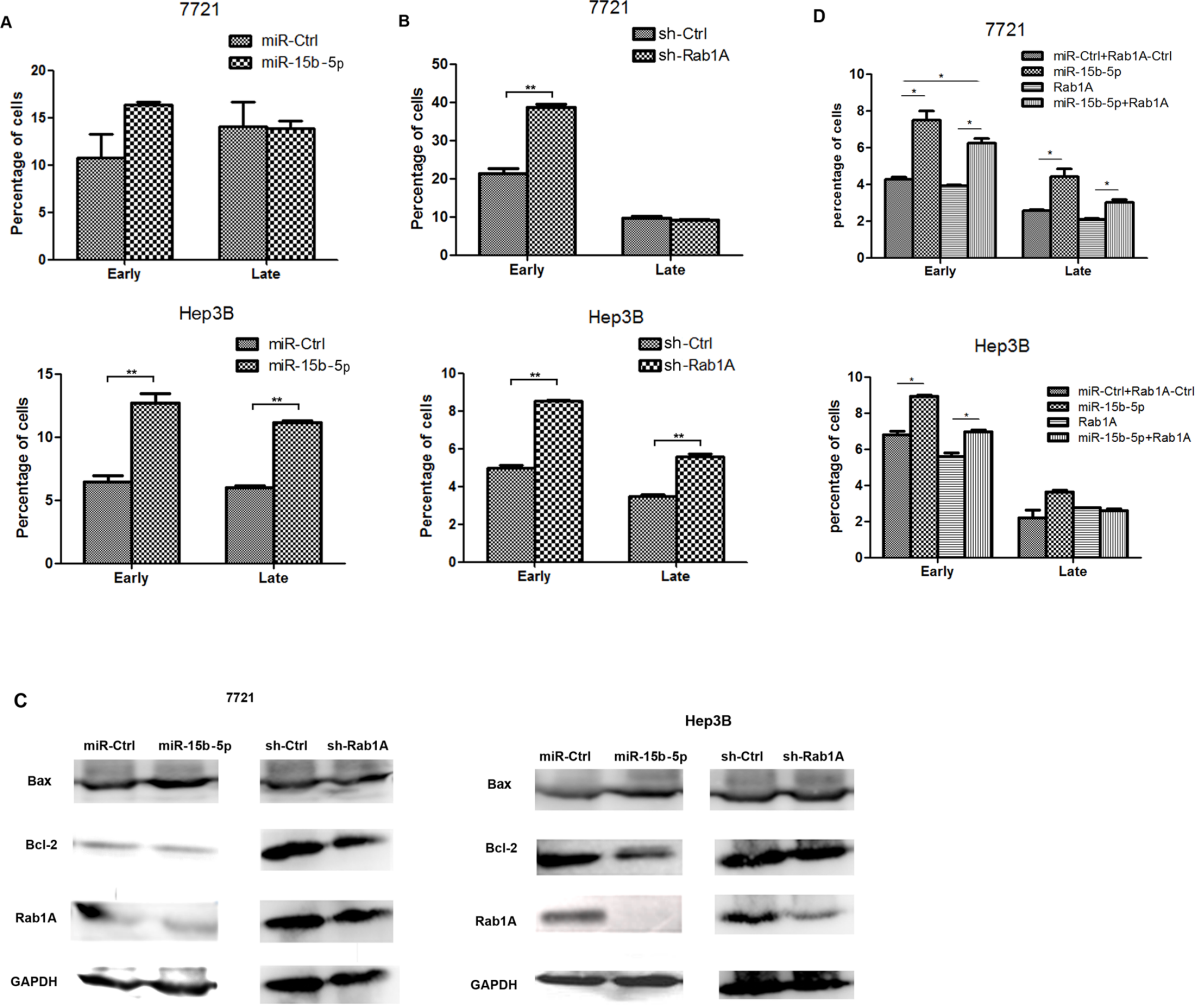
miR-15b-5p promoted HCC cell apoptosis by targeting and suppressing Rab1A **A.** miR-15b-5p was transfected in SMMC-7721and Hep3B cells for 48 hours. **B.** SMMC-7721 and Hep3B cells were transfected with shRab1A for 48 hours and apoptosis was determined by Annexin Vstaining and flow cytometry. **C.** Western blot analyses performed in SMMC-7721and Hep3B cells after infection of miR-15b-5p or shRab1A compare with their control vector, respectively. The relative protein expression levels of Rab1A, Bcl-2, Bax were measured, GADPH used as an internal control **D.** Overexpression of Rab1A reverses the promoted apoptosis roles of miR-15b-5p. Data represented mean values. (*P < 0.05; **P < 0.01.)

Apoptosis assay and western blot analysis were performed to determine if miR-15b-5p influences apoptosis in HCC cells by targeting and suppressing Rab1A. Expression in SMMC-7721 and Hep3B cells transfected with miR-15b-5p or sh-Rab1A was compared to the expression in cells transfected with the appropriate control. We found that overexpression of miR-15b-5p or silencing of Rab1A induced apoptosis in both SMMC-7721 and Hep3B cells (Figure 4A–B). Furthermore, Bcl-2 expression significantly decreased in HCC cells transfected with either miR-15b-5p or shRab1A, while the Bax expression increased (Figure 4C). Rab1A re-expression reversed the miR-15b-5p-induced apoptosis (Figure 4D). As shown in Supplementary Figure S2, the protein expression levels of Bax and Bcl-2 were assayed by Western blot analyses. These results indicated that miR-15b-5p induces apoptosis of HCC cells by suppressing Rab1A and this suppression involves changes in the expression of Bcl-2 and Bax. Upregulation of apoptosis is important in the treatment of cancer, particularly for inhibiting tumorigenesis.

### Inhibition of miR-15b-5p contributes to proliferation and suppresses apoptosis in HCC cells

**Figure 5:**
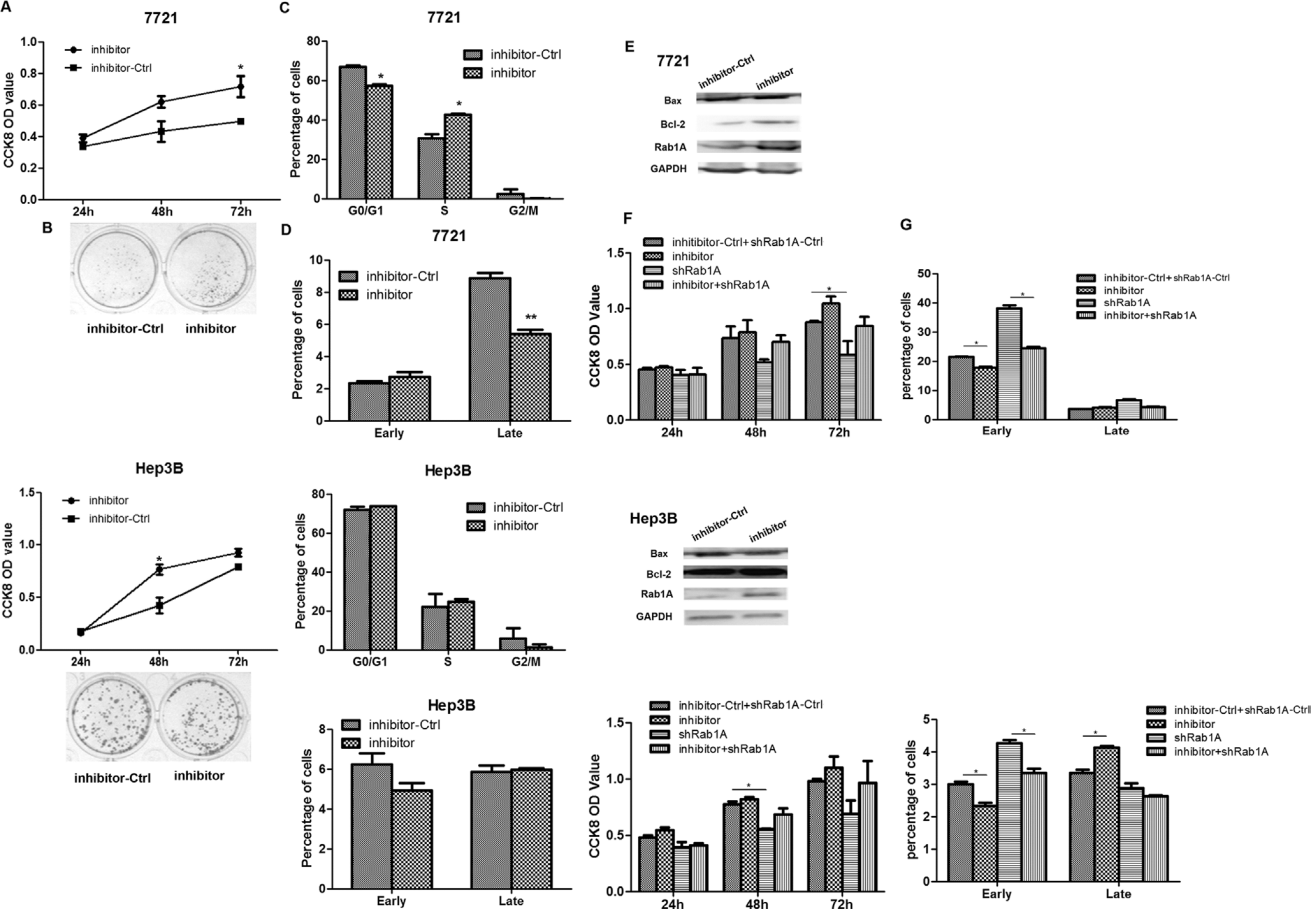
Inhibiting miR-15b-5p contributes to HCC cell growth **A.** CCK-8 assay was performed to detect the effects of inhibitor of miR-15b-5p on SMMC-7721 and Hep3B cell at 24, 48, and 72 hours after transfection. **B.** colony formation assay was performed at day 14 after transfection with inhibitor of miR-15b-5p or NC control, respectively. **C.** The representative histograms for cell-cycle distribution transfected with miR-15b-5p-inhibitor in both SMMC-7721 and Hep3B cells. Data represented the mean ± SEM (*p < 0.05; **p < 0.01). **D.** SMMC-7721 and Hep3B cells were transfected with inhibitor of miR-15b-5p or control for 48 hours and apoptosis was determined by Annexin Vstaining and flow cytometry. **E.** Western blot analyses measured in SMMC-7721 and Hep3B cells after transfection with the inhibitor of miR-15b-5p. The relative protein expression levels of Rab1A, Bcl-2, Bax were measured, GADPH served as a housekeeping control. **F–G.** Silencing the expression of Rab1A eliminated the proliferative effects of of miR-15b-5p-inhibitor and reverses the suppressed apoptosis roles of miR-15b-5p-inhibitor, Data represented mean values. (*P < 0.05; **P < 0.01).

The CCK8 assay indicated that cell growth increased in SMMC-7721 and Hep3B cells transfected with miR-15b-5p-inhibitor (Figure 5A). The result of the colony formation assay was consistent with the result of the CCK8 assay (Figure 5B). Cell cycle assay was performed to detect the percentage of cells in G1, S and G2 cell-cycle phases, that cells transfected with miR-15b-5p-inhibitor has less cell in G1 phase, more in S phase in SMMC-7721 cells, there is almost no change in Hep3B cells (Figure 5C). Furthermore, we performed apoptosis assay and found that suppressing miR-15b-5p slightly decreased the number of apoptotic cells compared with control treated SMMC-7721 cells. (Figure 5D). There was no detectable change in the Hep3B cells. In addition, the relative protein expression of Bax decreased, while the expression of Rab1A and Bcl-2 increased in cells transfected with miR-15b-5p-inhibitor (Figure 5E). Transfection of sh-Rab1A after transfection with inhibitor-ctrl or miR-15b-5p-inhibitor in SMMC-7721 or Hep3B cells reversed the miR-15b-5p-inhibitor-induced growth enhancement and resulted in apoptosis inhibition (Figure 5F-5G). As shown in Supplementary Figure S3, the protein expression levels of Bax and Bcl-2 were assayed by Western blot analyses. These data suggested that suppressing the expression of miR-15b-5p promotes the growth of HCC cells by targeting and suppressing Rab1A.

### miR-15b-5p induces ERS by suppressing Rab1A and decreases Bcl-2 in HCC cells

**Figure 6:**
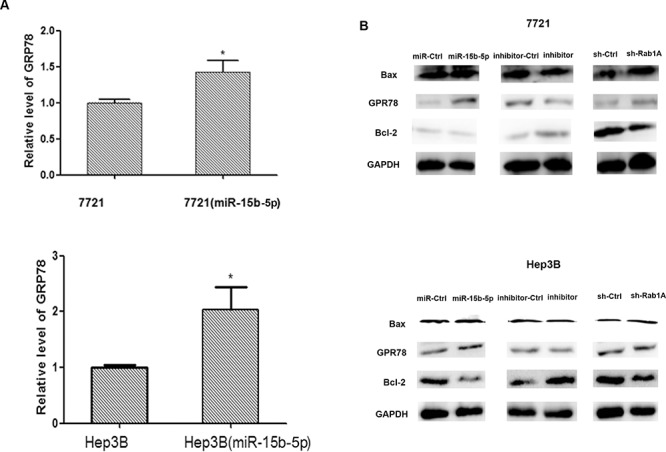
Overexpression of miR-15b-5p induces endoplasmic reticulum stress and results apoptotic death in human hepatocellular carcinoma **A.** qRT-PCR was used to determined the relative expression levels of GRP78 in SMMC-7721and Hep3B cells after transfection with miR-15b-5p over-expression vector for 48 h. **B.** Western blot analyses were performed to determine the expression of protein (GRP78, Bcl-2, bax) in SMMC-7721 and Hep3B cells after infection of miR-15b-5p or inhibitor of miR-15b-5p or ShRabA and compare with their respective control. GADPH served as an internal control. Each data represented mean ± SEM. (*p < 0.05; **p < 0.01).

To determine if miR-15b-5p-dependent Rab1A inhibition activates ERS, we monitored changes in the expression of glucose-regulated protein 78 (GRP78), which belongs to the group of ER molecular chaperones and is considered a marker of ERS. Results from quantitative RT-PCR suggested that GRP78 mRNA significantly increased in SMMC-7721 and Hep3B cells transfected with miR-15b-5p (Figure 6A); and the change of GRP78 protein was consistent with these results (Figure 6B). The protein expression of Bcl-2 was lower in miR-15b-5p-transfected and shRab1A-transfected cells compared with the expression in control transfected cells, while the expression of Bax was somewhat higher (Figure 6B). As shown in Figure 4C, we demonstrated that overexpression of miR-15b-5p decreased the protein expression of Rab1A in SMMC-7721 and Hep3B cells. These data suggest that miR-15b-5p targets and suppresses Rab1A, induces ERS, and results in apoptosis in HCC cells.

### miR-15b-5p inhibits HCC tumor growth *in vivo*

**Figure 7:**
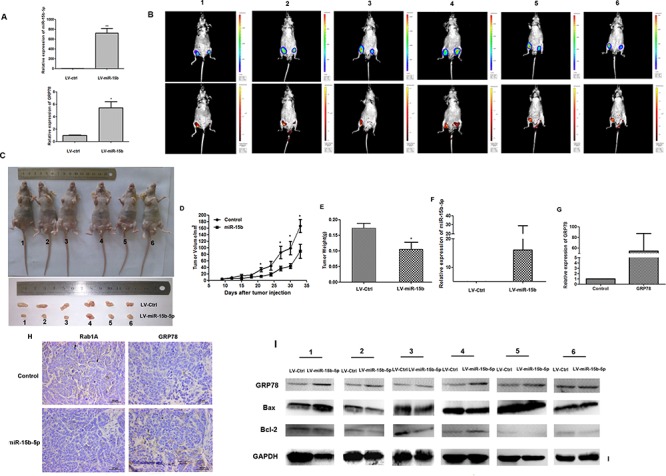
Overexpression of miR-15b-5p induces ERS and apoptotic death in HCC cells **A.** qRT–PCR were performed to determine the expression level of miR-15b-5p and GRP78 after SMMC-7721cells infected with LV-miR-15b and LV-CN (*P < 0.05, **p < 0.01, Student's *t*-test). **B.** Tumor volume at the 33 day after infection was assessed by small animal imaging analysis. The tumor volume detected by two methods (Luc and GFP). the right flank was injected with LV-miR-15b and left flank was injected with LV-CN in 6 nude mice, respectively. **C.** Gross morphology of tumors injected with either LV-miR-15b and LV-CN cells after 33 days. **D-E.** Tumor growth curves and tumor weight. **F-G.** qRT-PCR was used to measure the levels of miR-15b-5p and GRP78 expression in tumor xenografts. **H.** IHC staining of Rab1A/GRP78 in the tumor tissue from the mice injected with LV-miR-15b and LV-CN. **I.** The protein expression levels of Bcl-2, Bax, and GRP78 were measured by western blot in the tumor tissue from the mice injected with LV-miR-15b and LV-CN. GAPDH was served as a housekeeping control.

The expression of miR-15b-5p and GRP78 was detected by qRT-PCR in SMMC-7721 cells infected with LV- miR-15b (Figure 7A). To evaluate the role of miR-15b in the growth of HCC *in vivo*, we injected 1 × 10^6^ of LV-miR-15b-infected and LV-CN-infected SMMC-7721 cells subcutaneously into either posterior flank of the same nude mouse. Growth of the xenografts was measured every three days for 33 days. Tumor growth formed by LV-miR-15b was significantly smaller than that formed by the LV-CN, The tumor volume detected by two methods (Luc and GFP), as shown in Figure 7B-7C. Tumor growth curves show the growth of the LV- miR-15b-infected group is slower than growth of the control infected group (Figure 7D). The average tumor weight of the LV-miR-15b xenografts was 0.105g compared to the average weight of 0.173 g of LV-CN xenografts (Figure 7E). Expression of miR-15b-5p and GRP78 was measured by qRT-PCR of the RNA from LV-miR-15b–treated LV-CN-treated tumors. We found that miR-15b-5p and GRP78 were higher in LV-miR-15b-treated tumors than in LV-CN-treated tumors (Figure 7F-7G). This result is consistent with the results in the HCC cell lines. Results from immunohistochemistry also suggest that Rab1A expression is lower and GRP78 expression is higher in LV-miR-15b–treated tumors than in LV-CN-treated tumors. (Figure 7H). Western blot analysis showed that Bcl-2 expression is reduced while expression of Bax and GRP78 is increased in LV-miR-15b-treated tumors (Figure 7I). These results were consistent with the results in HCC cell lines. Based on these findings, we concluded that miR-15b-5p suppresses proliferation and induces apoptosis in HCC cells by suppressing Rab1A protein.

## DISCUSSION

The Rab GTPase protein family constitutes the largest family of small GTPases and contains more than 60 human Rab proteins. The family localizes to specific intracellular membranes and plays an important role in membrane trafficking of the ER, Golgi, endosomes, lysosomes, phagosomes, and autophagosome pathway. [22]. As a member of the Rab GTPase protein family, Rab1A is located at ER exit sites and at the pre-Golgi intermediate compartment. The best characterized function of Rab1A is to mediate ER–Golgi trafficking in later stages of ER-to-Golgi transport by facilitating the fusion of COPII coated vesicles with the cis face of the Golgi [23, 24].

The ER plays a major role in the synthesis, folding, and structural maturation of more than a third of all proteins made in the cell. Numerous genetic and environmental factors affect the ability of the cells to properly fold and post-translationally modify secretory and transmembrane proteins in the ER [25]. Misfolded or damaged proteins accumulated in this organelle can exceed the capacity of the ER to fold them and subsequently cause ER stress (ERS). When cells undergo ERS, they quickly restore protein-folding capacity in order to decrease the accumulation of misfolded proteins and activate UPR, an intracellular signaling pathway that allows cells to adapt to the changing environment and reestablish normal ER function by inducing transcriptional and translational silencing. [26]. However, if ERS persists at high levels, adaptive mechanisms are useless, ERS is irremediable, and UPR transforms into an alternate signaling platform called terminal UPR, promoting cell death.

ERS interferes with the inhibitory effects of the ER chaperone, Grp78, also known as heat shock protein A5 (HSPA5) or binding immunoglobulin protein (BiP). Transmembrane proteins PERK, IRE1 and ATF6 are activated through dissociation with GRP78 when unfolded proteins and chaperones is imbalanced [27]. UPR can transition into apoptosis with high expression of the CHOP/GADD153 transcription factor. Upregulation of CHOP/GADD153 promotes cell death and upregulates the expression of proapoptotic factors by restraining the expression of antiapoptotic BCL-2. When caspase 3 is activated, resulting in a Beclin 1 C-terminal cleavage product that translocates to the mitochondria, pro-apoptotic factors are released and apoptosis occurs. ERS induced apoptosis is related to multiple signaling pathways, including the caspase-12/caspase-9/caspase-3, PERK/ATF4/CHOP, IRE-1/ASK1/JNK, and p53 pathways [28–31].

We found an interesting phenomenon that miR-15b-5p expression was increased in HCC tissues; however, miR-15b-5p expression negatively correlated to HCC recurrence [21]. Although miR-15b-5p is increased in HCC, it is actually a tumor suppressor gene. Rab1A was previously reported to be an oncogene in human colorectal cancer [32], however, the effect on colon cancer apoptosis was not clear. Here, for the first time, our study reveals a possible relation between Rab, miRNA, and apoptosis. We demonstrate that miR-15b-5p suppresses Rab1A expression, thereby inducing ERS and apoptosis in HCC cells. These results may provide an important therapeutic strategy for fighting against cancer through ERS induced apoptosis.

## MATERIALS AND METHODS

### Cell lines and HCC tissues

Human liver cancer cell line SMMC-7721, HepG2, Hep3B and normal liver cell HL-7702 cells were obtained from the Key Laboratory of Environmentally and Genetically Associated Diseases at Xi'an Jiaotong University, Ministry of Education (Xi'an, Shaanxi, China). All the cells were cultivated in Dulbecco's modified Eagle's medium (PAA Laboratories, Pasching, Austria) supplemented with 10% fetal bovine serum at 37°C in 5% CO2. Twenty-eight paired hepatocellular carcinoma and matched normal tissues were obtained from patients who had undergone surgical gastric resection at the First Affiliated Hospital of Xi'an Jiaotong University College of Medicine (Xi'an, China). No local or systemic treatment had been conducted before surgery. The study was approved by the Institute Research Ethics Committee at Cancer, Xi'an Jiaotong University and all patients provided written informed consent.

### RNA extraction and quantitative real-time PCR

Total RNA, including miRNA from tissue samples and cells was extracted using TRIzol reagent (Invitrogen Life Technologies, Carlsbad, CA, USA). cDNA was synthesized from RNA, using a PrimeScript™ RT reagent Kit purchased from TAKARA (Takara Biotechnology Co., Ltd., Dalian, China), according to the manufacturer's instructions. The mature miRNA was reverse transcribed by miRNA-specific primers for quantification of miR-15b-5p, U6 served as a control. The reverse transcription primers of miR-15b-5p and U6 are listed in Table 1. Relative quantification of mRNA expression levels were performed using SYBR Premix Ex Taq II on an FTC-3000TM System (Funglyn Biotech Inc., Toronto, Canada) according to the manufacturer's instructions. U6 and GAPDH were used to normalize mRNA and miRNA level respectively. The relative expression of genes (miR-15b-5p, U6, GRP78, Rab1A, Gapdh) was calculated using the 2^−ΔΔCt^ method. The primers used were shown in Table 1 listed in Table 1.

**Table 1:** Primers

Name	Sequence (5′-3′)
U6- RT	RT:CGCTTCACGAATTTGCGTGTCAT
U6-F	Forward:GCTTCGGCAGCACATATACTAAAAT
U6-R	Reverse:CGCTTCACGAATTTGCGTGTCAT
miR-15b-5p-RT	RT:GTCGTATCCAGTGCGTGTCGTGGAGTCGGCAATTGCACTGGATACGACTGTAAAC
miR-15b-5p-RT-F	Forward:ATCCAGTGCGTGTCGTG
miR-15b-5p-RT-R	Reverse:TGCTTAGCAGCACATCATG
Rab1A3′UTR-S	CAAGCAGTCAGGTGGAGGTTGCTGCTAC
Rab1A3′UTR-A	TCGAGTAGCAGCAACCTCCACCTGACTGCTTGAGCT
Rab1A3′UTR-MS	CAAGCAGTCAGGTGGAGGTTGCGCATAC
Rab1A3′UTR-MA	TCGAGTATGCGCAACCTCCACCTGACTGCTTGAGCT
Rab1A-RT-F	Forward:GGGAAAACAATCAAGCTTCAAA
Rab1A-RT-R	Reverse:CTGGAGGTGATTGTTCGAAAT
gapdh-RT-F	Forward:AGCCACATCGCTCAGACAC
gapdh-RT-R	Reverse:GCCCAATACGACCAAATCC
miR-15b-S	GATGAATCCTACATTTTTGAG
miR-15b-A	AGTTTTGAATGAATTTCCTTA
Inhibitor-ctrl	TGACTGTACTGACTCGACTG
miR-15b-5p-inhibitor	TGTAAACCATGATGTGCTGCTA
GRP78-RT-F	Forward:AGCTGTAGCGTATGGTGCTG
GRP78-RT-R	Reverse:AAGGGGACATACATCAAGCAGT

### Construction of expression plasmids

The precursor of has-miR-15b was got by PCR from human liver cancer tissue, the primers were listed in Table 1. The sequence of Hsa-miR-15b is F5′ TTGAGGCCTTAAAGTACT GTAGCAGCACATCATGGTTTACATGCTACAGTCAA GATGCGAATCATTATTTGCTGCTCTAGAAATTTAAG GAAATTCAT3′ R Then human miR-15b precursor cloned into the EcoRI and HindIII sites of the pcDNA6.2-GW/EmGFP. While, the 3′ UTR of Rab1A. mRNA was constructed by synthetic oligonucleotides and inserted into the SacI and XhoI sites of the pmirGLO Dual-Luciferase miRNA Target Expression Vector (Promega). All the vector sequence information is provided in Table 1.

### miRNA and RNA interference

Hep3B cells and SMMC-7721 cells transfected with miR-15b, inhibitor, or shRab1A by Lipofectamine 2000 according to manufacturer's recommendation comparing with their control respectively. The inhibitor of miR-15b and inhibitor-control were synthesized in SangonBiotech (Shanghai, China). Silencing of Rab1A gene in SMMC-7721 cells and Hep3B cells was performed by shRNA transfection. shRNA targeting Rab1A (shRab1A), vector of re-expression Rab1A and their controls were purchased from GeneChem (Shanghai, China).

### miR-15b-5p directly targeted Rab1A

A fragment of the 3′-untranslated region (UTR) of Rab1A for miR-15b-5p was synthesized in SangonBiotech, (Shanghai, China), wild-type (Rab1A-WT) and mutant (Rab1A -MT) binding sites were cloned into the region of the pmiRGLO dual-luciferase reporter vector. The pmirGLO-Rab1A-3′UTR-wt or the pmirGLO-Rab1A-3′UTR-mut vector was cotransfected with miR-15b-5p into HEK293 cell lines using Lipofectamine 2000 (Invitrogen Life Technologies), pmirGLO vector as their control. HEK293 cells were obtained from the Key Laboratory of Environmentally and Genetically Associated Diseases at Xi'an Jiaotong University, Ministry of Education (Xi'an, China). Luciferase reporter gene assay was performed at 24 h post-transfection using the Dual-Luciferase Reporter Assay System (Promega Corporation, Madison, WI, USA) according to the manufacturer's protocol.

### Cell proliferation assay

Cell proliferation assay was performed at 24 h, 48 h, 72 h post-transfection according to the protocol of cell counting Kit-8(CCK8) (7Sea Biotech, Shanghai, China) according to the manufactocol. Absorbance was measured at a wavelength of 450 nm with FLUOstar OPTIMA (BMG).

### Colony formation assay

SMMC-7721, Hep3B cells were seeded in 6-well plates at 3000 cells/well after 24 hours of transfection, incubated for 2 weeks. Stained with 0.5% crystal viole for 30 min, The number of clones were counted by computer software (Bio-Rad quantity one).

### Cell-cycle analysis

SMMC-7721, Hep3B cells were seeded in 12-well plates at 50000 cells/well, washed with PBS and fixed in ice-cold ethanol at 4°C overnight, then washed twice again in PBS, added 150 μl 0.1 mg/ml Rnase A and 0.05 mg/ml propidium iodide (PI) for 30 minutes at 4°C. Cell-cycle distributions were detected by flow cytometer (FACSort; Becton).

### Cell apoptosis analysis

Cell apoptosis analysis was assessed using a Annexin-VFITC Apoptosis Detection Kit (Invitrogen) according to the manufacturer's instructions. SMMC-7721 and Hep3B were transfected with miR-15b-5p, miR-15b-5p-inhibitor, shRab1A, Rab1A compared with their control respectly when the cells had grown to 70–90% confluence. After transtection, cells were cultured at 37°C for 48 h. The cells were then stained using an Annexin V/fluorescein isothiocyanate Apoptosis Detection kit (Invitrogen Life Technologies) according to the manufacturer's instructions. Cell apoptosis were examined using a flow cytometer (Becton-Dickinson) and the apoptosis of cells was measured as the percentage by ModFite software.

### Western blot

For Western blot assay, the protein samples of tissue and transfected cells were lysed using RIPA buffer (Sigma-Aldrich). Lysates were then collected by centrifugation at 14, 000 × g for 20 min. Protein concentration was determined by a BCA protein assay kit. The equal amounts of protein lysates were run on 10% SDS-PAGE gels, and the separated proteins were then transferred to a methanol-activated PVDF membranes, blocked in 5% non-fat dry milk in Tris-buffered saline (pH 7.4) containing 0.1% Tween (TBST) for 2 h. Then, the primary antibodies Rab1A, GRP78 (Cell Signaling Technology, diluted 1:300), Bcl-2, Bax, GAPDH antibodies (Bioworld, diluted 1/500) were incubated over night at 4°C. Secondary anti-rabbit or anti-mouse (Pierce) were used on second day for 1 h at Room temperature. Protein expression was normalized to GAPDH levels in each sample.

### Tumorigenicity assay *in vivo*

The animal study protocol was reviewed and approved by the Institutional Animal Care and Use Committee of Xi'an Jiaotong University. Four-week-old male BALB/C nude mice were used to analyse tumorigenicity. SMMC-7721 cells were subcutaneously infected with 1 × 10^6^ LV-miR-15b and LV-CN in 100 μl PBS. Tumor size was measured by vernier caliper every 3 days for 33 days. Tumor volumes (V) were calculated according to the formula V = (L × w2) × 0.5, in which “L” is the length and “W” is the width of the tumor. *In vivo* bioluminescence imaging was obtained by the system of IVIS Spectrum (USA).

### Immunohistochemistry

The tumor tissues were fixed in 10% formalin buffer and made into paraffin sections, 4-μm thick, Sections were deparaffinized with xylene and hydrated using graded alcohol, antigen retrieval and blocking were then performed. All slides were incubated using the primary antibodies (Rab1Aand GRP78) overnight at 4°C, then incubation with secondary antibodies, Detection was performed by 3, 3′-diaminobenzidine(DAB) and hematoxylin. Finally, digital images were assessed by Leica Q550 image analysis system.

### Statistical analysis

All statistical analyses were performed using SPSS Statistics 13.0 (SPSS, Chicago, IL, USA). Data were presented as mean ± SEM of at least three independent experiments; Statistical significance of the differences between groups was calculated using the Student *t*-test. All tests were two-sides and *P* < 0.05 was considered as statistically significant. The linear correlation coefficient (Pearson's r) was used to explore the association between miR-15b-5p and Rab1A expression.

## SUPPLEMENTARY FIGURES


