# Supplementary material for: miR-15b-5p induces endoplasmic reticulum stress and apoptosis in human hepatocellular carcinoma, both in vitro and in vivo, by suppressing Rab1A
Source: Oncotarget. 2015 May 18;6(18):16227–38. doi: 10.18632/oncotarget.3970 (PMC4599266; doi:10.18632/oncotarget.3970)
Supplement: Supplementary file 1 [file oncotarget-06-16227-s001.pdf]

SUPPLEMENTARY FIGURES

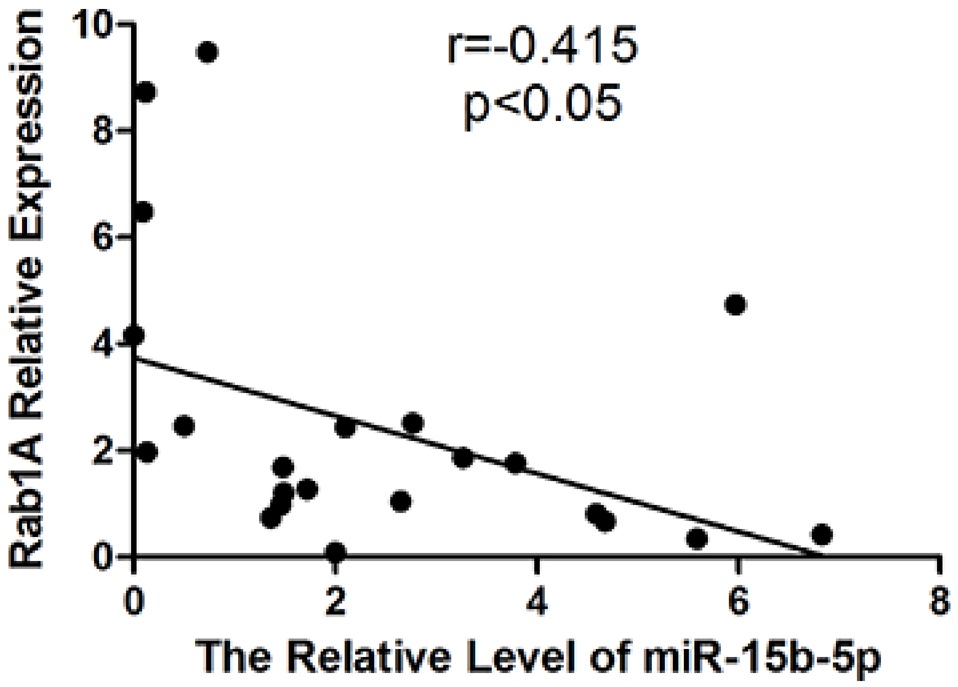

Supplementary Figure S1: There was a inverse correlation between miR-15b-5p and Rab1A mRNA expression levels. Expression of Rab1A analyzed by qRT–PCR and normalized to GAPDH. Expression of miR-15b-5p was examined by qRT–PCR analysis and normalized to U6 expression. Pearson's correlation coefficient was calculated to estimate the correlation between miR-15b-5p and Rab1A mRNA expression levels in HCC tissues and their normal control tissues.

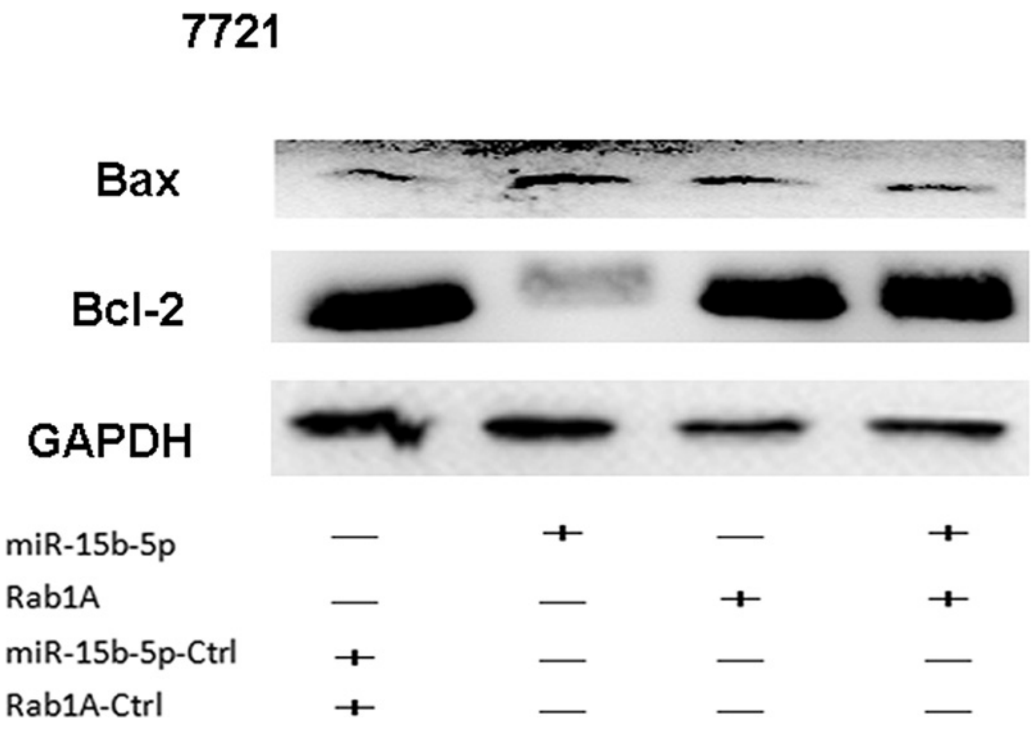

Supplementary Figure S2: Rab1A re-expression reversed the miR-15b-5p-induced apoptosis. SMMC-7721 cells were further transfected with the Rab1A expression vector or the control vector after transfection of miR-ctrl or miR-15b-5p. Western blot assay was performed at 48h post-transfection to detect the expression levels of Bax and Bcl-2. GADPH served as an internal control.

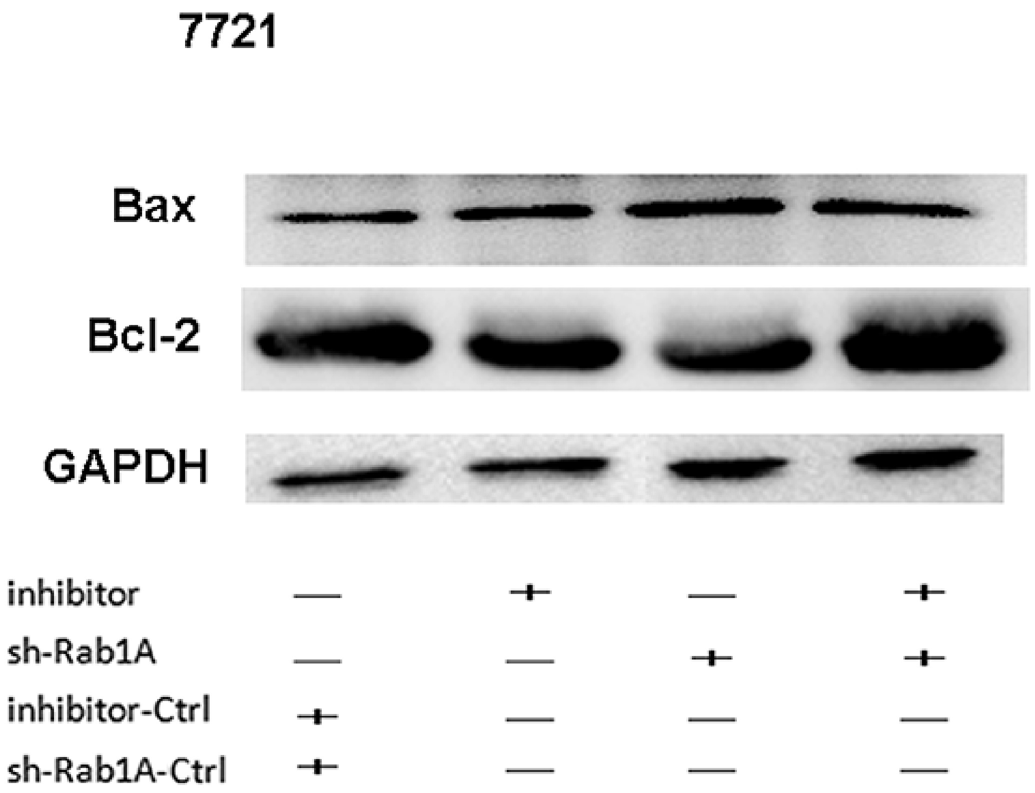

Supplementary Figure S3: Sh-Rab1A reversed the miR-15b-5p-inhibitor-induced apoptosis inhibition. SMMC-7721 cells were transfected with the sh-Rab1A expression vector or the control vector after transfection of miR-15b-5p-inhibitor or inhibitor-ctrl.The expression levels of Bax and Bcl-2 were analyzed by Western blot assay at 48h post-transfection. GAPDH was served as a housekeeping control.
